# Villous Tree Model with Active Contractions for Estimating Blood Flow Conditions in the Human Placenta

**DOI:** 10.2174/1874120701711010036

**Published:** 2017-04-14

**Authors:** Yoko Kato, Michelle L. Oyen, Graham J. Burton

**Affiliations:** 1 Faculty of Engineering, Tohoku Gakuin University, Tagajo, Miyagi, Japan; 2 Department of Engineering, University of Cambridge, Cambridge, United Kingdom; 3 Centre for Trophoblast Research and Development Physiology, Department of Neuroscience, University of Cambridge, Cambridge, United Kingdom

**Keywords:** Human placenta, Villous tree, Stem villi, Contraction, Placental function, Blood flow

## Abstract

**Background:**

In the human placenta, maternal and fetal bloods exchange substances through the surface of the villous trees: the fetal blood circulates in the villous trees, around which the maternal blood circulates. The blood flows directly influence fetal growth. Stem villi, the main supports of the villous tree, have contractile cells along the axes, whose contractions are expected to influence the blood circulations in the placenta. The displacement is neither measurable nor predictable while non-invasive measurements such as umbilical Doppler waveforms are helpful to predict the histological changes of the villous trees and vascularization in the placenta.

**Objective:**

The displacement caused by the contraction of the villous tree is necessary to predict the blood flows in the placenta. Hence, a computational villous tree model, which actively contracts, was developed in this study.

**Method:**

The villous tree model was based on the previous reports: shear moduli of the human placenta; branching patterns in the stem villi. The displacement pattern in the placenta was estimated by the computational model when the shear elastic moduli were changed.

**Results:**

The results show that the displacement caused by the contraction was influenced by the shear elastic moduli, but kept useful for the blood flows in the placenta. The characteristics agreed with the robustness of the blood flows in the placenta.

**Conclusion:**

The villous tree model, which actively contracts, was developed in this study. The combination of this computational model and non-invasive measurements will be useful to evaluate the condition of the placenta.

## INTRODUCTION

The human placenta has both maternal and fetal blood circulations, which exchange gases, nutrients and waste through the surface of the villous tree without mixture [1]: the maternal blood from the uterine arteries flows slowly into the intervillous space and returns to the uterine veins while the fetal blood from the umbilical arteries flows into arteries within the chorionic plate, circulates through blood vessels in the villous tree, and returns to the umbilical veins. The influence of the maternal and fetal blood flows on the transport of substances has been reported previously [2, 3].

The villous tree is composed of stem villi, intermediate villi, terminal villi and mesenchymal villi. Stem villi are the main support of the villous tree between the two bounding surfaces of the placenta. The stem villi have the contractile cells, which surround the arterial and venous vessels and run along the longitudinal axis of the branch [4-11]. The contraction of the stem villi has been observed *in vitro* [5, 12-14], which is expected to help the circulations of the maternal and fetal bloods. The maximum velocity of the contraction was much smaller than that of uterus, and the peak isometric tension was 1.39 kPa for electrical tetanus and 1.32 kPa for KCl exposure on average [14]. The contraction directly assists the fetal blood flow in the vessels of the stem villi because the contractile cells surround the vessels axially. However, the fetal blood in the capillary of the villous tree and the maternal blood in the intervillous space are not surrounded by the contractile cells directly, and similarity in the directions between the blood flow and the contractile cells has not been clear. In the meantime, the mechanical properties of the human placenta were evaluated by tensile, compression and shear [15]: The elastic moduli measured by shear were much smaller than those by tensile and compression. The shear stress was less than 1 kPa when the strain was 1 (strain velocity < 0.04 /s). Considering that the blood vessels were aligned with the direction of the force [15], the shear elastic moduli in the surroundings of the blood vessels would be much less than 1 kPa. Comparing the tension of the contractile cell with the shear elastic moduli, the displacement and its propagation in the placenta would occur although neither direct measurement nor prediction for the displacement is possible.

The conditions of the placenta, concerning the blood flow, are non-invasively observed by ultrasound and magnetic resonance (MR). The velocity of the blood flow in the umbilical artery is measured by ultrasound Doppler velocimetry and described as flow velocity waveforms (FVWs). The histological characteristics in the villous tree, including the distribution of the villous types and vascularization, can be estimated by FVW at end diastolic: positive, absent or reverse [16-18]. The oxygen environment, which is evaluated by oxygen-enhanced MRI and BOLD MRI [19], influenced the bifurcation pattern of the villous tree: hypoxia enhances the bifurcation [20, 21]. The magnitude of the perfusion in the placenta can be expressed as relative values by three-dimensional (3D) power Doppler [22] and contrast-enhanced MR images [23], but the direction is hardly measured.

In general, the normal mature placenta shows legions in some degree, such as infarctions, which can prevent the blood circulations in the placenta. 3D power Doppler indicated that the perfusion in the normal placenta was kept through the gestational ages, from 15 to 40 weeks [24]. In the meantime, the diameter, branching patterns and generation of the stem villi were previously reported [1, 25] so that a computational model of the villous tree with active contractions can be developed. If the displacement caused by the contraction is corresponding to the perfusion evaluated by 3D power Doppler or MRI, the direction of the blood flow can be estimated by changing the distribution of the shear elastic moduli as the pattern of the displacement in the placenta agrees with that of the perfusion. Assuming that the contraction force and shear elastic moduli of the region surrounding the stem villi are representative of the placental conditions, the results of the computation can be translated properly.

In this study, a computational model of the villous tree in the human placenta with active contractions was developed for estimating the blood flow condition. The shape of the stem villi was based on the previous reports [1, 25], and the surroundings of the stem villi were assumed as one continuum, where the displacement caused by the contraction propagated because the branching pattern of the villi around the stem villi, and the shape of the intervillous space, which is the space surrounded by the villi and the maternal blood passes through, are complicated. By using this model, it was examined whether or not the contraction could assist the fetal blood flow in the capillary and the maternal blood flow in the intervillous space. Moreover, the influence of the mechanical properties in the villous tree on the pattern of the displacement was also examined.

## COMPUTATIONAL MODEL

### Stem Villi

The stem villi are categorized into the three groups: truncus chorii, next to the chorionic plate; rami chorii, next to the truncus chorii; ramuli chorii, between the rami chorii and the basal plate [1, 25]. The diameter gradually becomes smaller from the chorionic plate to the basal plate, and branches are found in all the groups except the truncus chorii. The branching pattern of the ramuli chorii is not equally dichotomous. The generations of branches in the rami chorii and ramuli chorii are up to 4, and from 1 to 30, respectively. Table **1** shows the size and branching pattern in the stem villi model. In this model, the chorionic and basal plates, and the boundaries between the categories (truncus chorii, rami chorii and ramuli chorii) were parallel to each other. Firstly, how to use the centripetal and centrifugal orders at the branch to describe the branching pattern in Table **1** is explained. Figs. (**1a** and **1b**) show a simple branching pattern designated by the centripetal order and centrifugal order: the tip and trunk are designated as 1 and maximum, respectively, at the centripetal order, and vice versa at the centrifugal order. The centripetal order is used to evaluate dichotomy [26, 27]. The bifurcation ratio, *R_b_*, is defined as the following equation:

(1) $$R_{b}=\frac{N_{u}}{N_{u+1}}$$

where *N_u_* is the number of branches at the centripetal order *u*. If *R_b_* is 2, the branching is equally dichotomous. Assuming that *R_b_* is constant, *N_u_* is a geometrical series given as:

(2) $$N_{u}={R_{b}}^{\left(u_{\max}-u\right)}$$

where *u_max_* is the maximum centripetal order. Hence,

(3) $$\ln N_{u}=(u_{\max}-u)\ln R_{b}$$

*R_b_* of the branches was calculated by the method of least squares. Table **1** shows that *R_b_* in the rami chorii was 2, but that in the ramuli chorii was not equal to 2. These values indicate that the branching patterns in the rami chorii and ramuli chorii are equally and unequally dichotomous, respectively. The centrifugal order at the tip, *C_f_*, in Table (**1**), corresponds to the generation of the branches. While *C_f_* in the rami chorii was constant, that in the ramuli chorii was varied. That is because the branches in the ramuli chorii were not symmetric.

The diameter range in each category was as follows: truncus chorii, 900 – 3000 μm; rami chorii, 300 – 1000 μm; ramuli chorii, 50 – 500 μm [25]. Table **1** shows that the diameter ranges agree with the aforementioned one. The change of the diameter at the truncus chorii was much larger than those at the rami chorii and ramuli chorii. For the smooth connection between the truncus chorii and rami chorii, the derivative of the radius with respect to the *z* coordinate should be zero at the boundary. Hence, the following equation was used to determine the radius of the branch at the truncus chorii:

(4) $$r=r_{\max}-\frac{\left(r_{\max}-r_{\min}\right)}{z_{\mathrm{tr}}}\sqrt{\left\{{z_{\mathrm{tr}}}^{2}-(z-z_{\mathrm{tr}}{)}^{2}\right\}}$$

where *r_max_* and *r_min_* are the maximum and minimum radii in the truncus chorii, and *z_tr_* is the boundary between the truncus chorii and rami chorii (*z_tr_* = 2.9 mm). In the rami chorii, the radius was decreased as the distance along the axis was longer. The radius of the branch in the ramuli chorii became larger as the centripetal order increased. The radii of the branches at the connecting point were modulated as all the branches showed the same radius.

The branches in the rami chorii were equally dichotomous as well as symmetric, and 16 branches were connected to those in the ramuli chorii at the boundary. The branches in the ramuli chorii should be unequally dichotomous. For making unequally dichotomous branches, the 2D diffusion-limited aggregation (DLA) models [28] were made by the free-software, dla-nd, which was developed by the Dr. Mark J. Stock (http://markjstock.org/dla-nd/). Because the path between the branching points was not smooth in this model, the line between the branching points was set as an axis of a branch.

As Table (**1**) shows, the longest distance from the chorionic plate in the model was 24.5 mm. According to the previous reports, the thickness of the human placenta and chorioamniotic membrane, the surface of the placenta, were 25 mm [1] and 243 μm [29] on average, respectively. It is calculated that the distance between the chorionic and basal plates is 24.8 mm. The longest distance from the chorionic plate in the model was close to the calculated distance between the chorionic and basal plates, based on the previous reports [1, 29]. In the meantime, the cross section of the model, parallel to the chorionic plate, had the bounding rectangle, whose size was 23.8 mm × 22.6 mm (width × height). The previous report [1] indicated that the placenta at term, whose diameter was 220 mm, had 60 – 70 villous stems. The area based on this diameter is 3.80 × 10^4^ mm^2^ so that the average cross section of the villous trees is 5.42 × 10^2^ - 6.33 × 10^2^ mm^2^, whose corresponding diameter is 26 – 28 mm. The size of the cross section in the model was close to that based on the previous report [1]. Figs. (**1c**-**1e**) show the villous tree model developed in this research. The size was 34.8 × 34.8 × 24.5 [mm] (1200 × 1200 × 847 [pixels], 29 μm/pixel).The Cartesian coordinate system, whose *z* axis was perpendicular to the chorionic plate, was used to describe the position in the model. Its origin was also on the chorionic plate.

### Contraction Direction

The contractile cells run along the longitudinal axis of the branch [4-11]. Each point at the boundary surface between the stem villi and the surroundings has two tangential directions as Fig. (**2**) shows. The tangential direction, closer to the axis of the branch than the other, was decided as the contraction direction.

At the truncus chorii, the axis of the branch was parallel to the *z* axis and its radius was largely changed as Equation (4) shows. The angle between the *z* axis and tangential direction (*φ_o_*) and the differentiation of Equation (4) by *z* are as follows:

(5) $$\varphi_{o}=\mathrm{atan}(\frac{\mathrm{dr}}{\mathrm{dz}})$$

(6) $$\frac{\mathrm{dr}}{\mathrm{dz}}=\frac{r_{\max}-r_{\min}}{z_{\mathrm{tr}}}\frac{z-z_{\mathrm{tr}}}{\sqrt{z_{\mathrm{tr}}^{2}-(z-z_{\mathrm{tr}}{)}^{2}}}$$

Considering that the direction of the contraction at the axis of the branch was (0, 0, -1),

(7) $$\varphi_{o}=\pi +\mathrm{atan}(\frac{\mathrm{dr}}{\mathrm{dz}})$$

When the angle between the point at the surface and the *x* axis in the *xy* plane is *θ_o_*, the tangential direction is (*sinφ_o_cosθ_o_*, *sinφ_o_sinθ_o_*, *cosφ_o_*). Comparing the rami chorii with the truncus chorii at Table (**1**), the diameter change at the rami chorii was 25% of that at the truncus chorii. In addition, the rami chorii showed the range of the *z* coordinate, which was about 3.7 times larger than that at the truncus chorii. The change of the diameter at the rami chorii was much smaller than that at the truncus chorii. Hence, the tangential direction at the surface was parallel to the axis of the branch at the rami chorii. Because the change of the diameter was also small at the ramuli chorii, the tangential direction was determined in the same way.

### Displacement

As Figs. (**1c**-**1e**) show, assuming that the surroundings of the stem villi were one continuum in this model, the propagation of the displacement in the placenta was evaluated by the model. A wave equation is generally described as below:

(8) $$\rho \frac{\partial^{2}u}{\partial t^{2}}=\mu \nabla^{2}u+(\lambda_{l}+\mu )\nabla (\nabla \cdot u)$$

where ***u*** is displacement vector, *ρ* is density, *λ_l_* and *μ* are Lamé’s constant. *μ* also shows a shear elastic modulus. Generally, biological tissue is incompressible, so that the second term in Equation (8) is zero. Hence,

(9) $$\rho \frac{\partial^{2}u}{\partial t^{2}}=\mu \nabla^{2}u$$

Hence, the shear wave (transverse wave), whose propagation is normal to the vibration and carried out in solid, was evaluated in this computation. The displacement caused by the shear wave is described as follows [30]:

(10) $$u=\xi_{o}\cos(\mathrm{kr}-\mathrm{𝜛t})$$

where *ξ_o_* is the amplitude, *k* is the wave number (*k* = 2π/*λ*, *λ* is wave length), *r* is the distance from the surface of the stem villi, *t* is time, and *ω* is angular frequency. *ξ_o_* was 0.1 μm in all the computational conditions. The shear elastic modulus, *μ*, is described as follows [31]:

(11) $$\mu =\frac{{𝜛}^{2}}{k^{2}}\rho$$

Considering that *k* = 2π/*λ* and *ω*=2π*ν* (*ν*, frequency),

(12) $$\mu =\rho \lambda^{2}v^{2}$$

*ρ* was 1.0 × 10^3^ kg/m^3^ because of biological tissues [32]. ν was 1.0 Hz, and *λ* was 0.29, 0.58 or 1.45 mm (10, 20 or 50 pixels) in the computation so that the shear moduli were 8.41 × 10^-5^, 3.36 × 10^-4^ and 2.10 × 10^-3^ Pa. The displacement is attenuated by viscoelastic properties so that the maximum distance for the propagation was 1.45, 2.9 and 4.35 mm (50, 100 and 150 pixels). In order to simplify the problem, *t* was set for zero. That is, the computation did not consider the time effect on the displacement. The maximum distance for the propagation was not dependent on the position. *λ* was kept constant in the surroundings, or became longer as the distance from the surface of the stem villi was longer. In the latter case, *λ* was increased from 0.29 mm to 1.45 mm every one-third of the maximum distance from the surface. Hence, there were 12 conditions in this computation.

## RESULTS

### Characteristic Positions and Visualization

The displacement of the surroundings of the stem villi is described by the polar coordinate system (magnitude, *φ* (0° ≤ φ ≤ 180°) and *θ* (-180° ≤ θ < 180°)) because the coordinate system was useful to separate the displacement into its magnitude and direction. Fig. (**3**) shows the results when *λ* and the maximum distance for the propagation were 1.45 mm and 4.35 mm, respectively. Fig. (**3a**) shows that the displaced area was gradually increased as the *z* coordinate became larger. The long and steep slope was observed from the truncus chorii to the rami chorii. The same features were observed in all the computational conditions. Figs. (**3b**, **3c** and **3d**) show the mean and standard deviation (SD) of each parameter: magnitude, *φ* and *θ*. The mean and SD were calculated for all the points in the displaced area, whose magnitude was more than zero. When the displacement is perpendicular to the chorionic plate, the value of *θ* cannot be determined. Hence, such a critical point was not included for the calculation of the mean and SD in *θ*. Fig. (**3b**) shows that the SD normalized by the mean was calculated in order to evaluate the magnitude range of the displacement in each *z* coordinate. The peak of the normalized SD was observed around the boundary between the truncus chorii and rami chorii, which is named as *z_d_*. The mean and SD about the direction of the displacement in each *z* coordinate are shown in Figs. (**4c** and **4d**). As Fig. (**4c**) shows, the mean of *φ* was kept around 90 degrees, but the SD of *φ* indicated two peaks, whose positions are named as *z_φ1_* and *z_φ2_*, respectively. Fig. (**3d**) shows that the mean of *θ* slightly decreased around the boundary between the rami chorii and ramuli chorii, which is named as *z_θ_*, while the SD of *θ* was kept around 90°. These characteristic *z* coordinates, *z_d_*, *z_φ1_*, *z_φ2_*, and *z_θ_*, were observed at all the computations. Fig. (**4**) shows the images which visualizes the magnitude, *φ*, and *θ* in each characteristic *z* coordinate and the middle *z* coordinates of the truncus chorii (*z_t_*), rami chorii (*z_r_*), and ramuli chorii (*z_rl_*) under the same computational condition as Fig. (**3**) shows. The displaced area became larger as the *z* coordinate was increased. The magnitude of the displacement was almost kept constant at every *z* coordinate although the magnitude in the limited area at *z_d_* was high. The distributions of *φ* and *θ* were largely changed along the *z* coordinate as shown in Figs. (**3c** and **3d**) The visualization of the displacement and direction like Fig. (**4**) is useful to find out critical points and important points for analysis.

### Magnitude of the Displacement

As Fig. (**4**) shows, the magnitude of the displacement was kept almost constant except the magnitude was high near the stem villi at *z_d_*. 90% of the displaced area showed that the magnitude relative to the maximum one was less than 0.17 for the maximum distance for the propagation = 1.45 mm and λ increasing as the distance from the surface of the stem villi, and 0.06 for other results. The SD normalized by the mean was large at *z_d_*, but the high magnitude was in the limited area. Hence, the maternal and fetal blood circulations in the villous tree and intervillous space would be influenced by the constant distribution of the displacement in the placenta. Because the shape of the stem villi directly influences the displacement pattern, the model whose shape near the high magnitude is changed will be developed and used for the computation to examine whether or not such a high magnitude is inevitable.

### Direction of the Displacement (*φ* and *θ*)

**Fig. (5):**
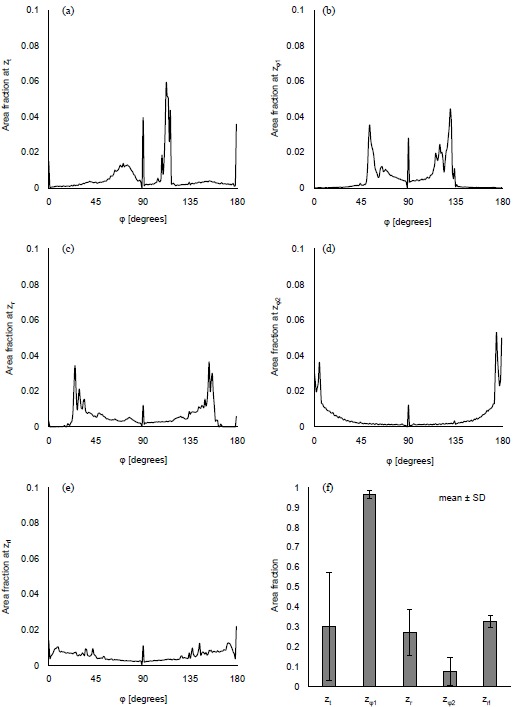
Distribution of *φ* in the characteristic positions for the displacement at *λ* = 1.45 mm and the maximum distance for the propagation = 4.35 mm. **(a-e)** show the distributions of *φ* at the following characteristic positions, *z_t_*, *z_φ1_*, *z_r_*, *z_φ2_* and *z_rl_*. The area fraction of *φ*, from 45° to 90°, for all the computation results is indicated in **(f)**.

Figs. (**5a**-**5e**) show the distribution of *φ* at the characteristic *z* coordinates (*z_φ1_*, *z_φ2_*) and the middle *z* coordinate in each category (*z_t_*, *z_r_*, *z_rl_*) for the same condition as shown in Fig. (**3**). The area fraction, which is the area for each *φ* normalized by all the displaced area, was used to evaluate the distribution of *φ*. The area fraction around *φ* = 90° (*φ* = 45° - 135°) was largest at *z_φ1_* (Fig. **5b**) and smallest at *z_φ2_* (Fig. **5d**). The same characteristics were observed in all the computations. For all the computational conditions, the mean and SD of the area fraction at *φ* = 45° - 135° were calculated at each *z* coordinate. As the average values in Fig. (**5f**) show, more than 90% of the displaced area showed *φ* from 45° to 135° at *z_φ1_*. The area with the same range of *φ* was around 10% at *z_φ2_*. Considering that the *φ* = 90° means the direction parallel to the *xy* plane, most of the displacement was parallel to the *xy* plane at *z_φ1_*, and parallel to the *z* axis at *z_φ2_*. Fig. (**5f**) shows that the mean of the area fraction at the other positions was around 0.3. Hence, *φ* did not have a preferred direction there. The displacement could help the maternal blood to go to the chorionic plate at *z_φ2_*, spread parallel to the chorionic plate and the basal plate at *z_φ1_*, and go toward the basal plate at *z_φ2_*. In the meantime, the branches in the stem villi around *z_φ1_* and *z_φ2_* had the axes largely different from the directions of the displacement there: the axis of the branch in the truncus chorii is perpendicular to the chorionic plate, and that in the rami chorii was largely changed because of its curvature. The displacements at *z_φ1_* and *z_φ2_* could assist the fetal blood to pass through the vessels in the stem villi. Moreover, the maternal and fetal bloods could be homogenized by the displacement at *z_t_*, *z_r_* and *z_rl_*. The SD was largest at *z_t_*, and smallest at *z_φ1_*. The SD at *z_rl_* was larger than that at *z_φ1_*, but much smaller than those at the other characteristic positions. Considering that *λ* and the maximum distance for the propagation are corresponding to the mechanical properties of the surroundings of the stem villi, *φ* at *z_φ1_* and *z_rl_* would be hardly influenced by the mechanical property of the surroundings, but *φ* at the truncus chorii would be vulnerable to it.

Figs. (**6a**-**6d**) show the distribution of *θ* at the characteristic *z* coordinates (*z_θ_*) and the middle *z* coordinate in each category (*z_t_*, *z_r_*, *z_rl_*) for the same condition as Fig. (**3**) shows. The area fraction, the area for each *θ* normalized by all the displaced area, was used for describing the distribution. Figs. (**6a** and **6b**) show that the similar distribution pattern was observed every 90° at *z_t_* and *z_r_*. The result agreed with the mean of *θ* at *z_t_* and *z_r_*, around zero. Fig. (**6c**) shows the distribution of *θ* at *z_θ_*, where the area fraction at *θ* = -180° - 90° (the third quadrant) and *θ* = 0° - 90° (the first quadrant) was larger than that at *θ* = -90° - 0° (the fourth quadrant) and *θ* = 90° - 180° (the second quadrant). This result agreed with the decrease of the mean at *z_θ_* in Fig. (**3d**). As Table (**1**) shows, the branches in the ramuli chorii were unequally dichotomous as well as asymmetric while those in the rami chorii were equally dichotomous as well as symmetric. Because the rami chorii had 16 branches connecting to those in the ramuli chorii, 16 types of the branching pattern were used in the ramuli chorii. *z_θ_* was located around the boundary between the rami chorii and ramuli chorii. The branches of the ramuli chorii at *z_θ_* were almost parallel to the *z* axis in the second and fourth quadrant, but those in the first and third quadrants were not. The angle of the branch at *z_θ_* would cause the characteristic distribution of *θ* at *z_θ_*. The similar distribution pattern was observed at *z_rl_*, but each peak was much smaller than that at *z_θ_* as Fig. (**6d**) shows. Because *z_rl_* was placed on the middle of the ramuli chorii, the feature caused at *z_θ_* would be weakened by the branches which showed various angles. All the computational results showed the same characteristics as Figs. (**6a**-**6d**) show. The SD of the area fraction in each interval (each value of *θ*) (*SD_in_*) was calculated in order to evaluate the uniformity of the distribution in *θ* for all the computations. Fig. (**6e**) shows that the average value of *SD_in_* at *z_θ_* was largest and that at *z_rl_* was smallest, among all the positions. The distribution of *θ* was most uniform at *z_rl_*, and most fluctuated at *z_θ_* in all the positions. The distribution of *θ* at *z_θ_* would be influenced by the angle of the branches in the ramuli chorii. Because *θ* at *z_rl_* did not have a preferred value, the maternal and fetal bloods could be homogenized. The SD values of *SD_in_* at *z_t_* and *z_θ_* was much larger than those of other two positions, and that at *z_rl_* was smallest in all the positions. The results show that the mechanical properties of the villus tree would strongly influence *θ* at *z_t_* and *z_θ_*, but hardly influence that at *z_rl_*. Even if the mechanical properties of the villous tree are changed, the homogenization of the bloods around the ramuli chorii would be kept.

## DISCUSSION

In this study, the computational villous tree model was developed, and used to evaluate the displacement in the human placenta caused by the contraction of the stem villi. The magnitude of the displacement was almost homogeneous, and the direction was useful for the fetal and maternal blood circulations. This tendency was maintained even if the mechanical properties of the placenta were changed. The experimental results such as MRI and 3D power Doppler angiography are described by scalar values so that the magnitude of the displacement could be directly compared with them. The resolution in the computation of this model was much higher than that in MRI or 3D power Doppler. Hence, representative values such as mean and SD will be necessary if the comparison between the experimental data and the computational result is carried out.

In the computation, every point in the stem villi contracted at the same time. Hence, the result in this study indicated that the displacement caused by the contraction would be helpful for the blood circulations when each contractile cell in the stem villi contracts at the same time. How to control the timing of the contraction for the effective blood circulation in the placenta can be investigated by this villous tree model. This investigation will be carried out in the future. The effect of the displacement on the maternal and fetal blood flows would be influenced by the directions of these blood flows. A computational model and method are necessary to investigate this effect. Developing them, with considering the usage of FWVs, will be the important topic to evaluate the blood circulation in the placenta.

## CONCLUSION

In this study, the computational model of the villous tree with active contractions was developed. The results based on this model show that the contraction could assist the maternal and fetal blood circulations in the placenta, and its effect would maintain even if the mechanical circumstances are changed. The combination between this computational model and non-invasive measurements will be useful to evaluate the condition of the placenta.

## Figures and Tables

**Fig. (1):**
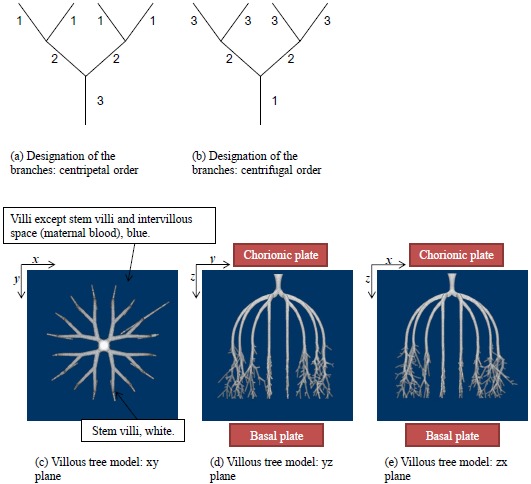
Villous tree model developed in this study. The branching pattern in the stem villi of the model was indicated by the centripetal and centrifugal orders at the branches, whose example is indicated by **(a)** and **(b)**, respectively. **(c-e)** show the side views of the model: the stem villi, white; the villi except the stem villi, blue; the intervillous space, which the maternal blood passes through, blue. As **(d)** and **(e)** show, this model is between the chorionic and basal plates. The *z* axis of the Cartesian coordinate system is perpendicular to the chorionic plate. The size of this model is 34.8 × 34.8 × 24.5 [mm] (1200 × 1200 × 847 [pixels], 29 μm/pixel).

**Fig. (2):**
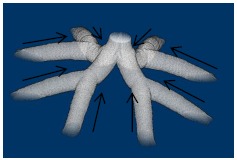
Contraction directions at the branches of the rami chorii. Arrows show the directions of the contractions. Each branch in the stem villi contracts along its axis and toward the junction.

**Fig. (3):**
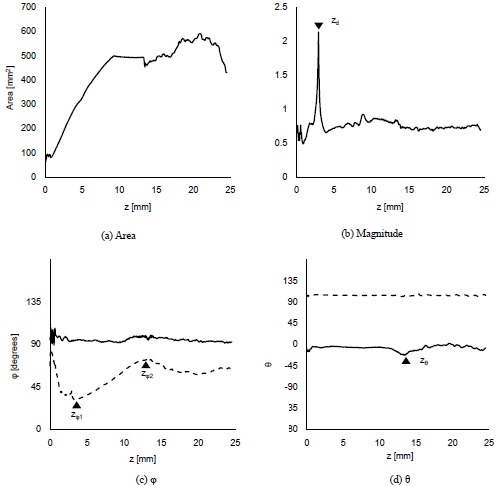
Displacement caused by the contraction of the stem villi. *λ* = 1.45 mm, and the maximum distance for the propagation = 4.35 mm. All the parameters at each *z* coordinate are shown. While **(a)** shows the displaced area, the magnitude **(b)** and direction (*φ*, *θ*) **(c, d)** of the displacement were described by the representative values, the mean and standard deviation (SD) for the displaced area. The solid and dotted lines in **(c)** and **(d)** are the mean and SD, respectively. The triangles show the characteristic positions, which were observed at all the computations. *z_d_*, *z_φ1_*, *z_φ2_*, and *z_θ_* are the *z* coordinates for these characteristic positions.

**Fig. (4):**
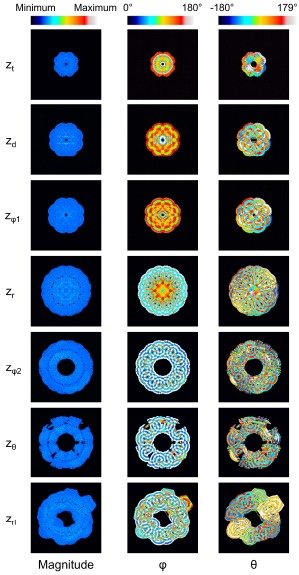
Displacement patterns in the characteristic positions. *λ* = 1.45 mm, and the maximum distance for the propagation = 4.35 mm. In addition to the characteristic positions in each parameter, *z_d_*, *z_φ_*_1_, *z_φ2_*, and *z_θ_,* the representative positions of the truncus chorii, rami chorii and ramuli chorii appear: *z_t_*, *z_r_* and *z_rl_*, which are the middle *z* coordinates of the truncus chorii, rami chorii, and ramuli chorii, respectively.

**Fig. (6):**
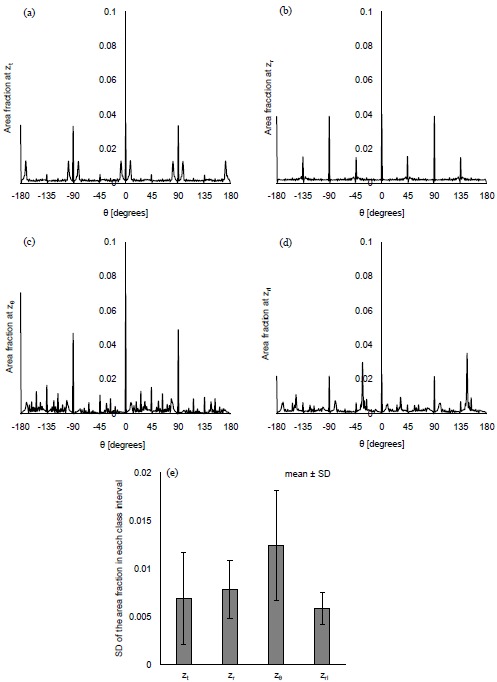
Distribution of *θ* for the displacement at *λ* = 1.45 mm and the maximum distance for the propagation = 4.35 mm. **(a-d)** show the distribution of *θ* in the characteristic positions, *z_t_*, *z_r_*, *z_θ_* and *z_rl_*. The SD of the area fraction in each class interval for all the computations (*SD_in_*) is indicated in **(e)**.

**Table 1:** Size and branching pattern in the stem villi model.

Parameter		Truncus chorii	Rami chorii	Ramuli chorii	
**Branch**	*d* [mm]	1 - 3	0.5 - 1	0.3 - 0.5	
	*z* [mm]	0 - 2.9	2.9 - 13.5	13.5 - 24.5	
**Axis**	*L_c_* [mm]	0 (*z* = 2.9)	8.99 (*z* = 13.5)	-	
	*L_b_*[mm]	-	0 (*z* =2.9)	*	
			0.29 (*z* = 3.9)		
			1.74 (*z* = 4.7)		
			4.64 (*z* = 5.8)		
	*r* [mm]	-	(*L_b_*< 1.74) 1.74	-	
			(*L_b_*≥1.74) 8.99		
	*R_b_*	-	2	2.22 - 6.02	
	*C_f_*	-	4	4 - 20	

The stem villi model was set in the space, whose size was 34.8 × 34.8 × 24.5 [mm] (*x* × *y* × *z*): the *z* coordinate shows the distance from the chorionic plate; *d*, the diameter of the branch; *L_c_*, the distance from the center of the space in each *z* coordinate (17.4 mm, 17.4 mm, *z*) to the axis of the branch at the boundary between the categories; *L_b_*, the distance from the center of the space in each *z* coordinate to the bifurcation; *, each branch bifurcated differently; *r*, the radius of curvature *R_b_*, bifurcation ratio; *C_f_*, the centrifugal order at the tip.

## References

[1] Benirschke K., Burton G.J., Baergen R.N. (2012). Pathology of the Human Placenta.

[2] Illsley N.P., Hall S., Stacey T.E. (1987). The modulation of glucose transfer across the human placenta by intervillous flow rates: An *in vitro* perfusion study. Trophoblast research.

[3] Lofthouse E.M., Perazzolo S., Brooks S., Crocker I.P., Glazier J.D., Johnstone E.D., Panitchob N., Sibley C.P., Widdows K.L., Sengers B.G., Lewis R.M. (2016). Phenylalanine transfer across the isolated perfused human placenta: an experimental and modeling investigation.. Am. J. Physiol. Regul. Integr. Comp. Physiol..

[4] Happe H. (1906). Beobachtungen an Eihäuten junger menschlicher Eier [Observation at embryonic membranes of early human embryos].. Anatomische Hefte.

[5] Krantz K.E., Parker J.C. (1963). Contractile properties of the smooth muscle in the human placenta.. Clin. Obstet. Gynecol..

[6] Graf R., Langer J.U., Schönfelder G., Öney T., Hartel-Schenk S., Reutter W., Schmidt H.H. (1994). The extravascular contractile system in the human placenta. Morphological and immunocytochemical investigations.. Anat. Embryol. (Berl.).

[7] Graf R., Schönfelder G., Mühlberger M., Gutsmann M. (1995). The perivascular contractile sheath of human placental stem villi: its isolation and characterization.. Placenta.

[8] Graf R., Neudeck H., Gossrau R., Vetter K. (1996). Elastic fibres are an essential component of human placental stem villous stroma and an integrated part of the perivascular contractile sheath.. Cell Tissue Res..

[9] Kohnen G., Kertschanska S., Demir R., Kaufmann P. (1996). Placental villous stroma as a model system for myofibroblast differentiation.. Histochem. Cell Biol..

[10] Graf R., Matejevic D., Schuppan D., Neudeck H., Shakibaei M., Vetter K. (1997). Molecular anatomy of the perivascular sheath in human placental stem villi: the contractile apparatus and its association to the extracellular matrix.. Cell Tissue Res..

[11] Demir R., Kosanke G., Kohnen G., Kertschanska S., Kaufmann P. (1997). Classification of human placental stem villi: review of structural and functional aspects.. Microsc. Res. Tech..

[12] Farley A.E., Graham C.H., Smith G.N. (2004). Contractile properties of human placental anchoring villi.. Am. J. Physiol. Regul. Integr. Comp. Physiol..

[13] Lecarpentier E., Claes V., Timbely O., Hébert J.L., Arsalane A., Moumen A., Guerin C., Guizard M., Michel F., Lecarpentier Y. (2013). Role of both actin-myosin cross bridges and NO-cGMP pathway modulators in the contraction and relaxation of human placental stem villi.. Placenta.

[14] Lecarpentier Y., Claes V., Lecarpentier E., Guerin C., Hébert J.L., Arsalane A., Moumen A., Krokidis X., Michel F., Timbely O. (2014). Ultraslow myosin molecular motors of placental contractile stem villi in humans.. PLoS One.

[15] Weed B.C., Borazjani A., Patnaik S.S., Prabhu R., Horstemeyer M.F., Ryan P.L., Franz T., Williams L.N., Liao J. (2012). Stress state and strain rate dependence of the human placenta.. Ann. Biomed. Eng..

[16] Todros T., Sciarrone A., Piccoli E., Guiot C., Kaufmann P., Kingdom J. Umbilical Doppler waveforms and placental villous angiogenesis in pregnancies complicated by fetal growth restriction..

[17] Todros T., Marzioni D., Lorenzi T., Piccoli E., Capparuccia L., Perugini V., Cardaropoli S., Romagnoli R., Gesuita R., Rolfo A., Paulesu L., Castellucci M. (2007). Evidence for a role of TGF-β1 in the expression and regulation of α-SMA in fetal growth restricted placentae.. Placenta.

[18] Todros T., Piccoli E., Rolfo A., Cardropoli S., Guiot C., Gaglioti P., Oberto M., Vasario E., Canigga I. (2011). Review: Feto-placental vascularization: a multifaceted approach.. Placenta.

[19] Huen I., Morris D.M., Wright C., Parker G.J.M., Sibley C.P., Johnstone E.D., Naish J.H. (2013). R_1_ and R_2_ *changes in the human placenta in response to maternal oxygen challenge. Magn. Reson. Med..

[20] Kaufmann P., Luckhardt M., Schweikhart G., Cantle S.J. (1987). Cross-sectional features and three-dimensional structure of human placental villi.. Placenta.

[21] Kingdom J.C.P., Kaufmann P. (1997). Oxygen and placental villous development: origins of fetal hypoxia.. Placenta.

[22] Raine-Fenning N.J., Campbell B.K., Clewes J.S., Kendall N.R., Johnson I.R. (2003). The reliability of virtual organ computer-aided analysis (VOCAL) for the semiquantification of ovarian, endometrial and subendometrial perfusion.. Ultrasound Obstet. Gynecol..

[23] Brunelli R., Masselli G., Parasassi T., De Spirito M., Papi M., Perrone G., Pittaluga E., Gualdi G., Pollettini E., Pittalis A., Anceschi M.M. (2010). Intervillous circulation in intra-uterine growth restriction. Correlation to fetal well being.. Placenta.

[24] Morel O., Grangé G., Fresson J., Schaaps J.P., Foidart J.M., Cabrol D., Tsatsaris V. (2011). Vascularization of the placenta and the sub-placental myometrium: feasibility and reproducibility of a three-dimensional power Doppler ultrasound quantification technique. A pilot study.. J. Matern. Fetal Neonatal Med..

[25] Kaufmann P., Sen D.K., Schweikhart G. (1979). Classification of human placental villi. I. Histology.. Cell Tissue Res..

[26] Oohata S., Shidei T. (1971). Studies on the branching structure of trees: I. Bifurcation ratio of trees in Horton’s law.. Jap. J. Ecol..

[27] Kosanke G., Castellucci M., Kaufmann P., Mironov V.A. (1993). Branching patterns of human placental villous trees: perspectives of topological analysis.. Placenta.

[28] Meakin P. (1998). Fractals, scaling and growth far from equilibrium (Cambridge nonlinear science series 5)..

[29] Oxlund H., Helmig R., Halaburt J.T., Uldbjerg N. (1990). Biomechanical analysis of human chorioamniotic membranes.. Eur. J. Obstet. Gynecol. Reprod. Biol..

[30] Muthupillai R., Lomas D.J., Rossman P.J., Greenleaf J.F., Manduca A., Ehman R.L. (1995). Magnetic resonance elastography by direct visualization of propagating acoustic strain waves.. Science.

[31] Madsen E.L., Sathoff H.J., Zagzebski J.A. (1983). Ultrasonic shear wave properties of soft tissues and tissuelike materials.. J. Acoust. Soc. Am..

[32] Burlew M.M., Madsen E.L., Zagzebski J.A., Banjavic R.A., Sum S.W. (1980). A new ultrasound tissue-equivalent material.. Radiology.

